# Factors Affecting Sleep Quality among University Medical and Nursing Students: A Study in Two Countries in the Mediterranean Region

**DOI:** 10.3390/diseases12050089

**Published:** 2024-05-05

**Authors:** Fadila Bousgheiri, Ali Allouch, Karima Sammoud, Rut Navarro-Martínez, Vanessa Ibáñez-del Valle, Meftaha Senhaji, Omar Cauli, Nisrin El Mlili, Adil Najdi

**Affiliations:** 1 Department of Epidemiology, Public Health, and Social Sciences, Faculty of Medicine and Pharmacy of Tangier, Abdelmalek Essaâdi University (UAE), Tangier 93030, Morocco; f.bousgheiri@uae.ac.ma (F.B.); sammoudkarima@gmail.com (K.S.); a.najdi@uae.ac.ma (A.N.); 2 Higher Institute of Nursing and Health Techniques of Tetouan (ISPITS-T), Tetouan 93000, Morocco; ali.allouch@etu.uae.ac.ma (A.A.); bioniss@hotmail.com (N.E.M.); 3 Department of Biology and Health, Faculty of Sciences, University Abdelmalek Essaâdi, Tetouan 93000, Morocco; msenhaji@uae.ac.ma; 4 Department of Nursing, Faculty of Nursing and Podiatry, University of Valencia, 46010 Valencia, Spain; rut.navarro@uv.es (R.N.-M.); maria.v.ibanez@uv.es (V.I.-d.V.); 5 Frailty and Research Organized Group (FROG), University of Valencia, 46010 Valencia, Spain

**Keywords:** sleep quality, Pittsburgh Sleep Quality Index, medical and nursing students, physical activity, smartphone addiction

## Abstract

Poor sleep quality, a global public health concern, poses a significant burden on individuals, particularly health care university students facing intense academic stress. A three-center cross-sectional study was conducted at the Higher Institute of Nursing and Health Sciences in Tetouan (Morocco), Faculty of Medicine in Tangier (Morocco) and Faculty of Nursing in Valencia (Spain). We collected various data using a sociodemographic questionnaire, the Pittsburgh sleep quality questionnaire, the international physical activity questionnaire (IPAQ) and the smartphone addiction questionnaire short-version (SAS-SV). A total of 1210 students were included in our study (mean age 20.4 years, 67.2% female, nursing students (66.2%) and medical students (33.8%), 76.1% students from Morocco and 33.9% from Spain). Analysis revealed a higher prevalence of poor sleep quality among Moroccans students compared to Spanish ones (*p* < 0.001), that nursing students showed less favorable sleep quality than medical students (*p* < 0.011) and that living with a chronic disease was linked to less favorable sleep quality (*p* < 0.001). Lastly, intense or weak physical activity and smartphone addiction were correlated with poor sleep quality (*p* < 0.001). In the multivariate analysis, an association persisted between poor sleep quality and factors such as the country of study (Odds ratio (OR): 6.25 [95% Confidence Interval (CI): 4.34–9.09]), involvement in nursing studies (OR: 3.50 [95% CI: 2.36–5.27]), and the presence of chronic diseases (OR: 2.70 [95% CI: 1.72–4.16]), (*p* < 0.01 each). Our findings highlight the multifaceted factors affecting sleep quality in young university students. The implications underscore the imperative of interventions tailored to this demographic group.

## 1. Introduction

Sleep is a global public health issue and a public health burden [1]. Poor sleep quality can have an impact on individuals of all ages and populations [1], and particularly health science students, whose academic careers are highly stressful [2]. A recently published umbrella review reported a prevalence of self-reported sleep problems in medical and nursing students between 40 and 55% [3,4,5,6,7,8]. The prevalence of sleep problems among medical students varies across countries; for example, in China, the prevalence seems to be as low as 25% [9,10], while Iran had the highest prevalence of around 58% [11]. Across the continents, poor sleep quality has been reported to be higher in Europe, followed by the Americas, Africa, Asia and Oceania [4]. Among health sciences students, nursing students displayed the highest prevalence of poor sleep quality [12]. 

Poor sleep quality leads to increased fatigue, greater irritability, daytime dysfunctions, slower reactions and the increased consumption of caffeine and alcohol [1,13]. In medical students, academic grades correlated significantly with sleep quality scores [14]. Conversely, the benefits of sleep for students include improved concentration, better memory, optimal mental and physical health, more effective decision-making and improved academic results [15,16]. Adequate sleep is essential for students’ overall well-being. Additionally, getting enough sleep can also help students manage stress and improve their mood, leading to a better overall quality of life. It can also improve problem-solving skills and creativity, as well as reducing the risk of accidents and injuries. In overall terms, emphasizing the importance of sleep is crucial for students to perform at their best academically and maintain their physical and mental health [15,17,18].

Several factors can influence sleep in the young population, including excessive use of electronic devices before bedtime [19,20,21]. Devices such as computers, smartphones and tablets can disrupt sleep due to the blue light they emit [22,23]. Smartphone addiction in medical students was reported by around 40% in both Asia and Europe [24,25,26] and positively correlated with poor sleep quality and with other mental health concerns such as stress, anxiety, depression, neuroticism and general health among Asian medical students [25]. Additional factors identified in the literature examining the relationship between physical activity and sleep quality have shown a range of correlations between the two items [27,28,29,30]. However, this association seem partly mediated by the intensity of physical activity, as a random effects meta-analysis showed that moderate-to-high intensity physical activity was associated with better sleep quality, while a weak negative association between moderate-to-vigorous physical activity level and sleep duration was also found [28].

Health issues and diseases including mental disorders can significantly impact the quality of sleep. Specific medical disorders and health conditions can result in difficulties with sleep, disturbances or changes in sleep patterns. Individuals with chronic illnesses often struggle to maintain quality sleep due to various factors related to their medical condition [31,32,33,34].

While studies have been conducted on various age groups, including older individuals, adults, children and adolescents, there is a lack of research specifically targeting young students. Furthermore, the literature on the factors influencing sleep in this population is limited. Our study aims to bridge this gap in the research by examining sleep problems specific to young students. Unlike previous studies, which have primarily examined determinants of sleep quality in other populations, our research specifically targets factors impacting sleep quality in young students such as physical activity levels, sedentary behavior and smartphone/tablet use, as these are known to affect sleep quality in this demographic group [29,35,36].

## 2. Materials and Methods

### 2.1. Study Design

Our research is a cross-sectional study conducted in three centers: the Higher Institute of Nursing and Health Sciences in northern Morocco (Tetouan), Faculty of Nursing at the University of Valencia, Valencia, Spain and the Faculty of Medicine and Pharmacy in northern Morocco (Tangier).

### 2.2. Target Population 

Our study targets university health sciences students in Morocco and Spain, including students registered in the degrees in Nursing or in Medicine. The inclusion criteria are as follows: the study included students studying the degree of Nursing or Medicine registered at the Higher Institute of Nursing and Health Sciences in northern Morocco, the Faculty of Nursing in Spain and at the Faculty of Medicine and Pharmacy in northern Morocco, irrespective of their specialization, who provided their consent to participate.

Random sampling stratified by institution (the Institute in Tetouan, the Faculty of Medicine and Pharmacy in Tangier and the Faculty of Nursing in Valencia) was used. The sample size was calculated on the basis of official data provided by the three centers. The total number of students enrolled in the different levels was 350, 950 and 1080, respectively. Thus, we applied the following sample calculation formula:

*n*_0_ = (z)^2^ p (1 − p)/d^2^ *n*_0_ = 384

where d denotes the margin of error, which is 5%, Z denotes the confidence level, p is 95% and heterogeneity is 50%.

To calculate the size (n) for a population of limited size, we will use Cochran’s corrected formula, as follows:

n = 331

response rate of 20%.

N = n + (n × 0.2) = 398 students

Based on the total number of students in each center, the minimum number of students required was 58 students from the Higher Institute of Nursing and Health Sciences in Northern Morocco (Tetouan), 159 students from the Faculty of Medicine and Pharmacy in northern Morocco (Tangier), and 181 students from the Faculty of Nursing at the University of Valencia, Valencia, Spain. 

### 2.3. Study Questionnaires

In this study, we gathered diverse data using a case report form that included questions about sociodemographic information, sleep patterns, physical activity and smartphone addiction data. The volunteers in the study were given this self-administered form in French. The initial section gathered sociodemographic details, including age, sex, education level, medical history and history of psychoactive substance use. We also used three additional questionnaires in both their French and Spanish versions: the Pittsburgh Sleep Quality Questionnaire [37,38,39,40,41,42], the iPAQ Physical Activity Questionnaire [43,44], and the SAS-SV Smartphone Addiction Questionnaire [45].

#### 2.3.1. Assessment of Sleep Quality

We used the Pittsburgh Sleep Quality Index (PSQI), a 19-item self-report questionnaire, to assess sleep quality. It assesses seven sleep components: (1) sleep quality, (2) sleep latency, (3) sleep duration, (4) habitual sleep efficiency, (5) sleep disturbance, (6) use of sleeping pills and (7) daytime dysfunction. Each component is rated on a scale from 0 to 3. The total score derived from the sum of these components ranges from 0 to 21, with higher scores indicating poorer sleep quality, and an overall score of more than 5 indicating a “poor” sleeper [37]. We used the French version with a Cronbach’s alpha of 0.93 [41] and the Spanish version with a Cronbach’s alpha of 0.805 [40,42]. 

#### 2.3.2. Assessment of Physical Activity

Total physical activity was measured using the short version of the IPAQ [46,47]. This is a seven-question self-report questionnaire designed to assess types and intensity of physical activity and daily time spent sitting; it is validated in both French and Spanish, with good Cronbach’s alpha scores of 0.84 and 0.93, respectively [43,44]. The participants were asked to recall their activities over the previous week, and provide information on their walking, moderate-intensity activities, vigorous-intensity activities and time spent sitting [46,48].

#### 2.3.3. Smartphone Addiction

Smartphone addiction was assessed using the short version of the Smartphone Addiction Scale (SAS-SV) [49]. This self-report questionnaire consists of 10 questions and is divided into six subscales. We used the translated and validated French version for students in Morocco and the Spanish version for students in Spain, with good Cronbach’s alpha scores of 0.90 and 0.88, respectively [45]. The total score on the scale ranges from 10 to 60, with the highest score indicating a higher level of “smartphone addiction”. According to the proposal by Kwon and colleagues in 2013, individuals with scores of 31 and 33 (out of 60) are classified as “excessive smartphone users” for men and women, respectively.

### 2.4. Statistical Analysis

Version 25 of the Statistical Package for the Social Sciences (SPSS) software (SPSS Inc., Chicago, IL, USA) was used for all the analyses. Descriptive statistics were used to describe quantitative variables, such as age, BMI and scale scores, which were presented using means and standard deviations, while qualitative variables, such as gender, country of study and study field, were reported as percentages. A Student’s t-test for independent samples was employed for the univariate analysis of the continuous variables, and the Chi-squared test was used for the categorical variables. A logistic regression model was applied to examine independent and interactive associations between smartphone addiction, physical activity and sleep quality.

### 2.5. Ethics Considerations

The research was approved by the Oujda Biomedical Research Ethics Committee (CERBO) with number 07/2022, and the Human Research Ethics Committee of the University of Valencia (Spain) (procedure number 2298864, 13 October 2022). All the participants were informed that their participation was entirely voluntary, and that they had the right to refuse to participate and to withdraw from the study at any time without any penalty. They were also informed that they had the right to ask questions and resolve any doubts they might have from the outset and throughout the study. Confidentiality and anonymity were guaranteed throughout the study. Additionally, the participants received sufficient information about the purpose of the study and the procedures before signing the informed consent form, and they were assured that their answers would remain confidential. They were also informed about the contents of their file.

## 3. Results

### 3.1. Overall Sample Descriptive Characteristics

We enrolled 1,210 students in our study. The majority were young, with an average age of 20.43 +/− 2.11. There was a predominance of women (67.2%, CI 95%) [64.5–69.9] and a high percentage of nursing students (66.2%, CI 95%) [63.5–68.9]. The average Pittsburgh score was 7.02 +/− 3.3, indicating that an estimated 74% (CI 95%) [71.3–76.7] of the students experienced poor sleep quality. Additionally, 61.9% (CI 95%) [57.4–66.2] of the students reported engaging in intense physical activity. The results also indicated that 53.7% (CI 95%) [50.8–56.6] of the students did not have a smartphone addiction, with an average score of 30 +/− 10 (Table 1).

### 3.2. Descriptive Analysis of Student Education by Country

Another descriptive analysis of the main variables studied across the two countries revealed that 76.1% (CI 95%) [73.6–78.5] of the Moroccan students and 23.9% (CI 95%) [21.5–26.4] of the Spanish students exhibited certain characteristics. The data also indicated a higher prevalence of poor sleep quality among the Moroccan students (81.7%, CI 95%) [78.7–84.3] compared to the Spanish students (54.0%, CI 95%) [48–59.8]. Additionally, the results showed that a smaller percentage of the Spanish students (31.1%, CI 95%) [25.8–36.8] reported smartphone addiction compared to the Moroccan students (51.2%, CI 95%) [47.8–54.5] (Table 2).

### 3.3. Factors Associated with Sleep Quality

**Figure 1:**
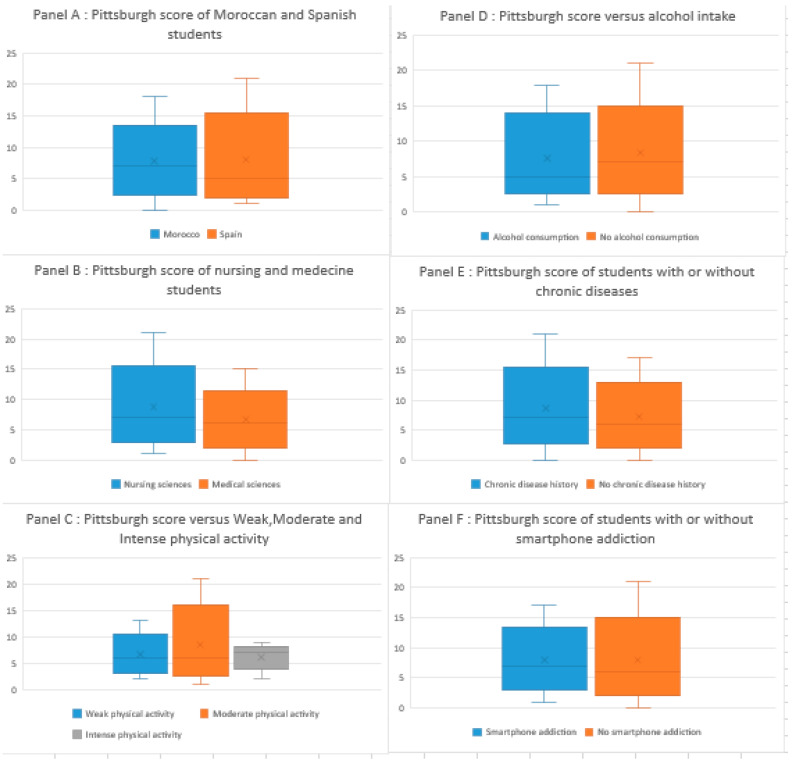
Pittsburgh scores across various student groups and factors.

The univariate analysis revealed significant associations between the country of study and sleep quality, indicating a higher prevalence of poor sleep quality among the Moroccan students (81.7%) compared to their Spanish counterparts (54%). Similarly, the field of study showed a significant correlation, with nursing students experiencing less favorable sleep quality than medical students. Alcohol consumption was also significantly associated with better sleep quality, while chronic diseases were linked to less favorable sleep quality. Lastly, physical activity and smartphone addiction were correlated with poor sleep quality, with an increased prevalence of smartphone addiction and intense physical activity among those with poor sleep quality. Interestingly, no significant associations were observed between sleep quality and gender or tobacco consumption (Table 3, Figure 1).

We performed a multivariate analysis to determine the specific impact of each factor on sleep quality, taking out confounding factors.

The initial model incorporates the factors found to be statistically significant in the univariate analysis and factors with a *p*-value of 0.2, resulting in a significant association between Moroccan nationality, nursing studies and the presence of chronic diseases. However, no significant association was observed between smartphone addiction, physical activity and sleep quality (Table 4).

## 4. Discussion

Our study reported a higher prevalence of poor sleep quality in nursing and medical students in two culturally different countries of the Mediterranean region. Previous studies among young students have indicated low levels of good sleep quality, and our study confirms this public health concern, with 74% of students experiencing poor sleep quality, compared to 26% experiencing good sleep quality [15,46,47,48,49,50]. Different factors are associated with poor sleep quality in bivariate analyses, such as students’ country of education, their field of study being nursing versus medicine, alcohol consumption, the presence of comorbid chronic conditions, a history of chronic disease, the intensity of their physical activity and whether they experience smartphone addiction. The most powerful associations found in the multivariate analysis (as shown in Table 4) were with the country of education, the study field, e.g., nursing versus medical students and the presence of chronic diseases. 

The comparison between Morocco and Spain revealed notable differences, as the Moroccan students exhibited a higher prevalence of sleep disorders than their Spanish counterparts. This variation was also observed in other factors affecting sleep quality, such as high smartphone addiction rates among Moroccan students, intense physical activity practiced by 79.3% of Moroccans and a higher incidence of chronic illness at 24.3% among the Moroccans, compared to 17.6% among Spanish students. These disparities raise important questions about the potential influence of cultural and environmental factors on the sleep quality of university students [51]. Recent studies have shown that sleep is influenced and shaped by cultural factors, including cultural values, beliefs and practices. Finally, most of the research includes countries in Asia, Europe, Australasia and North America, and very few cross-cultural studies have been conducted in countries of Africa. A review of the causes of poor sleep quality in African young adults indicates that poor sleep quality is higher in Africa than other continents [52]. According to two studies, black people are more likely to have sleep problems, which raises the likelihood of poor sleep quality in Africa [53,54,55]. Poor sleep quality among university students is also higher in Africa than in other studies, because of the different genes involved in circadian/sleep activity [56,57].

Although some studies have reported female vulnerability to poor sleep quality [49,50], in our study, sex differences were not found; however, it may be considered that statistical limitation could have played a role in this finding, since females were overrepresented in the sample. However, characteristic profiles of sleep duration differed between female and male university students, and the prevalence of concurrent psychological alterations known to affect sleep quality, such as the level of anxiety, depressive symptoms or stress, were not analyzed [50].

The comparison of medical and nursing students showed significant differences in sleep quality. In specific terms, 76.0% of the nursing students reported lower sleep quality, compared to 68.1% of the medical students. These differences may be linked to the specific academic demands of each field or to the different duration of the two degrees (3 or 4 years in Nursing versus 6 years in Medicine), suggesting the need for further investigation [16,48,58,59,60,61].

Concerning the role of tobacco consumption, no significant association between smoking and poor sleep quality was found. Conversely, Bogati et al. found that poor sleep quality is common among undergraduate students, and the consumption of caffeine, cigarettes and alcohol is associated with an increase in poor sleep quality among them [9,46]. The difference between our results and those in the literature could be due to the heterogeneity of our sample and variations in prevalence and substance consumption habits within our study population, which includes both Spanish and Moroccan students.

Concerning alcohol consumption, the bivariate analysis showed that students with no alcohol consumption had a higher prevalence of poor sleep quality than students who consumed alcohol-containing drinks. Historically, alcohol has been used as a sedative [62,63,64], and, therefore, the results of our study may be because a low intake of alcoholic beverages in the evening may help students to fall asleep, due to their hypnotic effect. Studies have found that, in non-alcoholics who consume alcohol occasionally, both high and low doses of alcohol initially improve sleep, but high doses of alcohol may cause sleep disturbance during the second half of the night-time sleep period [65]. On the other hand, these results are consistent with research conducted with university students [66], who found that alcohol consumption was associated with a 55% lower probability of poor sleep efficiency. Hence, individuals who drank alcoholic beverages had fewer insomnia symptoms than those who did not. However, in the literature, we found mixed results for studies that have examined the relationship between alcohol consumption and sleep quality in university students. In this way, other studies performed in university students from different countries found no significant association between alcohol and sleep parameters [67,68,69]. On the other hand, studies have also reported that excessive alcohol consumption is associated with poorer sleep quality [70,71]. The relatively low (2.8%) prevalence of alcohol use in the Moroccan student sample may have been insufficient to establish a statistically significant association with poor sleep quality in this country.

Chronic health conditions are associated with poor sleep patterns and less sleep overall [72,73,74]. These include chronic lung diseases, asthma, acid reflux, kidney disease, cancer, fibromyalgia and chronic pain [31,32,33,34,75]. Unfortunately, as with stress and anxiety, poor sleep quality can exacerbate the symptoms and discomfort felt with these conditions [2,76,77]. We found a confirmatory association between chronic diseases and the quality of sleep [78]. A recent study performed in university students who had a chronic disease or a family member with a chronic disease showed significantly higher frequencies of “high” level of stress, “poor sleep quality” and clinical insomnia, compared to those who did not [79]. This observation substantiates the findings in various earlier studies [78,80], which postulated that insufficient sleep significantly contributes to the onset and progression of chronic diseases. In emphasizing sleep disorders as an augmented risk factor in the twenty-first century, our results are consistent with contemporary research [73].

It is important for students with chronic health conditions to prioritize good sleep hygiene and to seek treatment for any sleep disturbances they may be experiencing. This may include working with healthcare providers to manage pain, discomfort and other symptoms that may be disrupting their sleep, as well as implementing healthy sleep habits such as maintaining a consistent sleep schedule, creating a relaxing bedtime routine and creating a comfortable sleeping environment [15,76]. In overall terms, for students with chronic health conditions, addressing sleep disturbances is an important aspect of managing their overall health and well-being [81].

Our findings reveal a significantly negative correlation with intense physical activity, which is consistent with some previous studies indicating a deterioration in sleep quality at higher levels of activity [82]. Importantly, the literature also includes research findings consistent with our results [27,30,83]. However, the lack of association with other levels of physical activity underlines the need for further research in this specific area, since it is well established that regular physical activity of a moderate intensity can improve sleep quality, reduce the time taken to fall asleep and improve sleep in general [29,84,85].

Our research also supported the association between smartphone addiction and sleep quality. This finding is aligned with a growing trend in the literature emphasizing the adverse impact of electronic devices on sleep, particularly among young adults [22,23,36,86,87,88].

This study found the prevalence of smartphone addiction to be 46.3%, using the SAS-SV (Smartphone Addiction Scale—Short Version), with rates of 31.1% among the Spanish students and 51.2% among the Moroccans. However, studies using the same measure Smartphone Addiction Scale (SAS) in other countries reported the following lower levels of smartphone addiction among young people: 29.8% in China [89], 33.1% in Brazil [90], 39.8% in Turkey [35] and 44.6% in Lebanon [91]. Other studies revealed high levels of prevalence among Malaysian (46.9%) [92] and Saudi (67%) students [16]. The high prevalence of smartphone addiction among the participants could be linked to the increasing rate of smartphone use among young people, including students, due to the widespread availability of Internet access in Morocco [93,94]. Based on the study design, we cannot infer about the bidirectional association between excessive phone usage and psychological disorders, wherein problematic phone use can contribute to the development of psychological disorders such as poor sleep quality, and conversely, pre-existing sleep problems can lead to problematic phone use [25,95]. Recently, the term nomophobia has been used to describe a psychological condition when people have a fear of being detached from mobile phone connectivity, and it is very difficult to differentiate whether the students become nomophobic due to mobile phone addiction or existing anxiety disorders manifested [58,96]. It can be postulated that a reciprocal relationship exists between phone addiction and poor sleep quality, forming a cyclical pattern with reinforcing effects. In a similar manner, several studies have demonstrated a bidirectional relationship between physical activity and sleep quality, as measured using actigraphy or recorded using sleep diaries [59,60,61].

Psychological interventions designed to improve sleep in university students [51,52] are as effective as those reported for general adult populations [53], in a similar way to pharmacological interventions commonly used in primary care [54]. Cognitive behavioral therapy for insomnia (one of the main problems of poor sleep quality in students) is the first-line psychological treatment recommended for insomnia by leading scientific associations [55,56,81]. The various interventions consist of a multicomponent treatment package that typically consists of sleep hygiene education, sleep restriction, stress reduction techniques such as relaxation, cognitive therapy to address unhelpful beliefs about sleep and techniques to help people cope effectively with their worries. Interestingly, mobile phones can be used to help in these interventions, as demonstrated in a trial for college students, who participated in a brief and personalized online sleep education intervention, which improved sleep behaviors, sleep quality and depression scores [57].

### Strengths and Limitations

There are several limitations that need to be considered when interpreting our findings. First, our cross-sectional design is vulnerable to biases, such as residual confounding and reverse causality. Additionally, our study’s reliance on questionnaire-based responses makes it susceptible to both recall and memory biases.

The strengths of our study are its inclusion of a large and diverse sample, enabling us to study multiple factors and increase the power of statistical tests. Another important point is that the study was carried out outside the students’ examination period in order to eliminate factors that could confound the results, such as exam-related stress, long hours spent studying in the evening and the use of stimulants.

## 5. Conclusions

In conclusion, the study provided valuable insights into the factors affecting sleep quality among medical students from two culturally different countries. The findings indicated a higher prevalence of poor sleep quality among Moroccan students compared to their Spanish counterparts. Importantly, a robust association was identified between engagement in nursing studies and poorer sleep quality. Furthermore, the study underscored a significant association between the presence of chronic diseases, intense physical activity and smartphone addiction with sleep quality, which all presented a significant link to a deterioration in sleep quality. In the multivariate analysis, a notable correlation persisted between poor sleep quality and factors such as the country of study, involvement in nursing studies and the presence of chronic diseases. These results highlight the importance of considering these factors in analytical studies aimed at enhancing sleep quality among students, with potential implications for public health interventions and educational support programs. 

## Figures and Tables

**Table 1:** Students’ characteristics.

Variables	Frequencies (%) N = 1210
Students’ country of education	
Morocco	76.1% (921)
Spain	23.9% (289)
Sex	
Male	32.8% (396)
Female	67.2% (812)
Study field	
Nursing students	66.2% (801)
Medical students	33.8% (409)
Tobacco consumption	
No	95.4% (1151)
Yes	4.6% (55)
Alcohol consumption	
No	83.9% (1013)
Yes	16.1% (194)
History of chronic disease	
No	77.4% (912)
Yes	22.6% (267)
Physical activity	
Weak	11.9% (58)
Moderate	26.2% (128)
Intense	61.9% (302)
Smartphone addiction	
No	53.7% (637)
Yes	46.3% (549)
Global sleep quality	
Good sleep quality	26% (273)
Poor sleep quality	74% (779)

**Table 2:** Profile of students by country of education.

Variables	Spain % (n = 289)	Morocco % (n = 921)	*p*-Value
Sex			
Male	13.5% (39)	38.8% (357)	*p* < 0.0001
Female	86.5% (250)	61.2% (562)	
Study field			
Nursing students	100% (289)	55.6% (512)	*p* < 0.0001
Medical students	-	44.4% (409)	
Tobacco consumption			
No	92% (299)	96.5% (885)	*p* < 0.001
Yes	8% ( 23)	3.5% (32)	
Alcohol consumption			
No	41.9 % (121)	97.2% (892)	*p* < 0.0001
Yes	58.1% (168)	2.8% (26)	
History of chronic disease			
No	82.4% (238)	75.7% (674)	*p* = 0.025
Yes	17.6% (51)	24.3% (216)	
Physical activity			
Weak	14% (15)	11.3% (43)	*p* < 0.001
Moderate	86% (92)	9.4% (36)	
Intense	_	79.3% (302)	
Smartphone addiction			
No	68.9% (199)	48.8% (438)	*p* < 0.0001
Yes	31.1% (90)	51.2% (459)	
Global sleep quality			
Good sleep quality	46.0% (133)	18.3% (140)	*p* < 0.0001
Poor sleep quality	54.0% (156)	81.7% (623)	

**Table 3:** The association between sleep quality and different factors: a univariate analysis.

Factors	Category	Quality of Sleep		*p*-Value
		Good Sleep Quality	Poor Sleep Quality	
Students’ country of education	Spain	46.0%	54.0%	*p* < 0.001
	Morocco	18.3%	81.7%	
Sex	Female	25.0%	75.0%	*p* = 0.09
	Male	28.4%	71.6%	
Study field	Nursing students	24.0%	76.0%	*p* = 0.011
	Medical students	31.9%	68.1%	
Tobacco consumption	No	26.1%	73.9%	*p* = 0.18
	Yes	20.9%	79.1%	
Alcohol consumption	No	22.5%	77.5%	*p* < 0.001
	Yes	42.2%	57.8%	
History of chronic disease	No	29.7%	70.3%	*p* < 0.001
	Yes	12.9%	87.1%	
Physical activity	Weak	27.6%	72.4%	*p* < 0.001
	Moderate	38.6%	61.4%	
	Intense	13.5%	86.5%	
Smartphone addiction	No	31.0%	69.0%	*p* < 0.001
	Yes	19.9%	80.1%	

**Table 4:** Poor sleep quality and associated factors: insights from multivariate analysis.

Factor	Initial Model		Final Model	
	Adjusted OR, CI 95%	*p*-Value	Adjusted OR, CI 95%	*p*-Value
Students’ country of education				
Spain	1		-	
Morocco	5.55 [3.33–8.33]	<0.001	6.25 [4.34–9.09]	*p* < 0.001
Sex				
Female	1.35 [0.83–2.20]	0.230	-	
Male	1		-	
Study field				
Nursing students	4.43 [2.30–8.52]	<0.001	3.50 [2.36–5.27]	*p* < 0.001
Medical students	1		-	
Tobacco consumption				
No	1		-	
Yes	1.87 [0.51–7.14]	0.285	-	
Alcohol consumption				
No	1		-	
Yes	0.97 [0.44–2.08]	0.823	-	
History of chronic disease				
No	1		-	
Yes	2.70 [1.40–5.26]	<0.003	2.70 [1.72–4.16]	*p* < 0.001
Physical activity				
Weak	1.98 [0.88–4.44]	0.094	1.96 [0.86–4.35]	0.106
Moderate	1.85 [0.70–4.25]	0.147	1.85 [0.81–4.17]	0.143
Intense	1			
Smartphone addiction				
No	1		-	
Yes	1.39 [0.82–2.37]	0.220	1.39 [0.82–2.35]	0.215

## Data Availability

The data presented in this study are available on request for scientific purposes from the corresponding author.
